# INTERNET AND HEALTH INFORMATION TECHNOLOGY USE AMONG MIDDLE-AGED AND OLDER ADULTS WITH ARTHRITIS

**DOI:** 10.1093/geroni/igae098.2461

**Published:** 2024-12-31

**Authors:** Sarah Lieber, Jerad Moxley, M Carrington Reid, Sara Czaja

**Affiliations:** Hospital for Special Surgery, New York, New York, United States; Weill Cornell Medicine, New York, New York, United States; Weill Cornell Medicine, Weill Cornell Medicine, New York, United States; Weill Cornell Medicine/Center on Aging and Behavioral Research, New York, New York, United States

## Abstract

Arthritis is a leading cause of healthcare utilization in older adults. Healthcare delivery increasingly occurs virtually. We assessed health information technology (HIT) use in middle-aged/older adults with and without self-reported arthritis by analyzing 2022 National Health Interview Survey data. Prevalence of internet access and internet use for health information, communication with doctor’s office, and review of test results was compared between adults ≥50 years of age with (versus without) arthritis (i.e., arthritis, rheumatoid arthritis, gout, lupus, fibromyalgia). Logistic regression was used to examine the relationship between arthritis and internet access and HIT behaviors. Among adults with arthritis (N=6371) for whom relevant survey data were available, 2640 (83%) reported internet access, and 1617 (61%), 1180 (45%), and 1359 (52%) used the internet for health information, communication with doctor’s office, and review of test results. Among adults without arthritis (N=9268) for whom relevant survey data were available, 4150 (88%) reported internet access, and 2367 (57%), 1687 (41%), and 2023 (49%) used the internet for health information, communication with doctor’s office, and review of test results. Arthritis was significantly associated with internet access and internet use for health information, communication with doctor’s office, and review of test results (all p< 0.01); the relationship between arthritis and internet use for health information, communication with doctor’s office, and review of test results remained significant after adjustment for age and sociodemographic characteristics (all p< 0.01). Further study of factors impacting HIT use in middle-aged/older adults with arthritis is needed to improve healthcare delivery in this population.

